# Hospital and Institutional News

**Published:** 1914-10-17

**Authors:** 


### HOSPITAL AND INSTITUTIONAL NEWS.

### CUMBERLAND INFIRMARY, CARLISLE.

As we go to press a further letter from Dr. Lediard reaches us, which confirms our view that a special investigation and independent report by a hospital expert of wide experience is desirable.

### THE MORAL OF THE TRAGEDY AT BARNET INFIRMARY.

The sad tragedy at Barnet Infirmary, where the superintendent nurse committed suicide, owing, as was stated in evidence at the inquest, to the interference in her work by individual Guardians and by the workhouse master, will be fresh in the memory of many of our readers. When the question of appointing her successor came before the board at their last meeting a letter was read from Dr. Stewart, the medical officer, suggesting that the board should define clearly and accurately the position of the superintendent nurse, relative to the master and matron. "Until she receives," he said, "her proper place, which has not been the case in the past, it is likely that friction and misunderstanding will exist between house officials and the infirmary—a condition of things detrimental to efficient and harmonious working." The practical solution of the position which he suggested was: "That a special book be provided, to be in the charge of the superintendent nurse, in which the nurses should enter all complaints, requests for off-duty time, and all such matters that should be brought to the notice of the infirmary officers, the superintendent nurse to deal with those matters that are within her province. For the rest, the superintendent nurse to bring these entries to the notice of the master or matron, or myself, according to the nature of the entry." Dr. Stewart's proposals, which were adopted by the board, are undoubtedly valuable, but friction is bound to recur until the infirmary is entirely separated administratively from the workhouse.

### ADVANCED CONSUMPTIVES AND KITCHENER'S ARMY.

What he describes as "a great crime" is detailed by Dr. W. H. Symons, medical officer of health for Bath, in a recent report to the sanitary committee, concerning the action of certain patients who had been under treatment in the city beds at Winsley Sanatorium. The report stated that three patients discharged themselves before their period of treatment expired, thus causing financial loss to the city through their beds being unoccupied, without benefit being received by the ratepayers. One patient, an advanced case, liable to severe hæmorrhage, had, with another similarly conditioned man, his expenses paid to a sanatorium, which, apparently because the weather was bad, both left after three days on a carrier's cart. They then launched complaints against the institution, which, the medical officer states, investigation by the committee showed not to have sufficient excuse. Both, he adds, had been under his observation for many years, and one had been much helped by friends and employees, including the treatment of his child at the public expense and of his family by church almoners. Since leaving the sanatorium, however, he refused to attend the tuberculosis dispensary, has been erratic in the work found for him, and liable to attacks of hæmorrhage at it. "I am informed," proceeds Dr. Symons, "that he has recently enlisted in Lord Kitchener's Army, no doubt receiving a bounty. I consider this a great crime."

### CONSUMPTIVE RECRUITS IN MILITARY HOSPITALS.

The next passage in Dr. Symons' report deserves a separate paragraph, so important are the questions which are raised. "Recently, when visiting a military hospital of 100 beds established in connection with mobilisation, the only cases I saw were eight patients suffering from tuberculosis. These had been sent into the hospital within a few days of embodiment. Such cases must be a serious encumbrance, engaging the attention of persons whose time is very valuable just now. I do not know how these cases pass the medical examiners, who are now mostly general practitioners—perhaps because it is impossible to give sufficient time to each case, it being supposed that the candidates will give correct information concerning their previous history. The patient's failure to do so is at the present time almost criminal. We have been accustomed to this kind of behaviour with some Poor-Law cases. One man, for example, has been admitted into the Frome Road Infirmary no fewer than forty-one times since notification of Poor-Law cases commenced in 1909. I cannot say what expense he has cost the city for disinfection, but he now usually gives a false address on leaving and we are generally unable to trace him." The committee decided that the case of the man who had joined Kitchener's Army be reported to the military authorities. He would be a source of serious infection to other recruits, and it is high time that the validity of the medical examination should be assured by absence of unnecessary hurry. The means by which this can be secured are described in our second leading article this week.

### IMPROVEMENTS AT THE NORTH EVINGTON INFIRMARY.

We are glad to find that one of the defects in the North Evington Infirmary, to which Sir Henry Burdett drew attention in his Report in The Hospital of May 30 last, is likely soon to be a thing of the past. At a meeting of the Leicester Board of Guardians, the board recently decided, with only one dissentient, to erect an operating room at a cost of £550. The board at the same time also decided to increase the accommodation for nurses and servants, to build a hospital block for 100 children and ten nurses, to provide a clinical room and a large recreation room, and to connect the hospital blocks by means of a corridor. The estimated cost of these improvements is £20,000. The work is to be proceeded with at once, and it is hoped that material help will thus be given to the local building trade, which, it is feared, will suffer from slackness of work in the coming winter.

### A HOSPITAL'S LOCAL REPUTATION.

The competition of the London hospitals with county institutions is probably less heard of now than ten years ago, but apparently some provincial hospitals still have to complain in this respect. That is the construction, at any rate, invited by the action of the chairman of the Royal Bucks Hospital, Aylesbury, Mr. G. Carrington, who has of late been promoting the interest of local bodies in the standard of his institution. With this end in view he recently received a deputation representing the Bradwell and Wolverton Good Samaritan Society, who were shown over the hospital by the chairman and members of the staff. At the conclusion of their inspection Mr. Wylie, on behalf of the deputation, expressed their thanks, and said that not only would they certainly be able to speak favourably of what they had seen, but if they could persuade their committee to use the Royal Bucks Hospital for cases coming within their purview he thought they could do so with every confidence. Another speaker promised the same assistance, and Mr. Carrington, in reply, remarked that they were now in a position to deal with difficult surgical and other cases equally as well as London hospitals, it therefore seemed a pity to send cases out of the county. If the deputation could assist in North Bucks, he added, the management would appreciate it, and the first-class motor ambulance soon to be available would enable them to deal with cases in every part of the county.

### LEEDS MEMORIAL TO DR. E. F. TREVELYAN.

A portrait of the late Dr. E. F. Trevelyan, formerly physician to the Leeds Infirmary, who died in December 1911, was presented by his colleagues and friends to the infirmary on Friday afternoon last week. The memorial consists of a portrait-bust, executed in bronze relief by Mr. Wyon, of London, and it is placed in the entrance hall of the infirmary, which already contains Chantrey's statue of the first William Hey, and other memorials of those who have rendered distinguished service to the hospital. There were present a considerable company, including most of the honorary and resident staff, the members of the weekly board, and other friends and subscribers. The memorial was unveiled by Dr. Trevelyan's sister, Mrs. Hicks, wife of the Bishop of Lincoln, and Miss Margaret Trevelyan, of Letchworth, was also present. Dr. A. G. Barrs, in making the presentation on behalf of the subscribers, dwelt on the long and faithful services rendered by the late Dr. Trevelyan to the infirmary and to other public institutions in this city. For twenty years he was physician to the general hospital, and as Professor of Pathology in the University of Leeds he did more than any other man to establish in Leeds the scientific study of this subject. He was the chief founder of the Leeds Association for the Prevention of Tuberculosis. He served the Leeds Public Dispensary and the Leeds Institute, and took a prominent part in the establishment of the Leeds Army Medical Corps. Into all his public work he threw himself with all his heart and energy. And yet, added Dr. Barrs, it is not simply this record of service that makes us offer this memorial. We are swayed by our remembrance of Edmund Trevelyan's personality. He was always an English gentleman, brave and true to the last, never more so than when he was suffering from his last painful illness. It is altogether a fit and proper thing that his memorial should be placed in the General Infirmary at Leeds. Mr. Charles Lupton, in accepting the gift on behalf of the infirmary, endorsed all that Dr. Barrs had said as to the value of Dr. Trevelyan's long public services in this city. Amongst those present were: Mr. Geo. Brown, Mr. Fred R. Spark, Mr. W. W. Lupton, Mr. W. H. Kitson, Mr. Fred J. Bray, Mr. Grosvenor Talbot, and Mr. Wyon, the sculptor. Dr. Eddison, Dr. A. G. Barrs, Dr. Wardrop Griffith, Dr. Hellier, Dr. G. Watson, Mr. Edward Ward, and many others, including members of the Army Medical Corps, were also at the ceremony.

### A THRILLING STORY OF THE LOSS OF THE "ABOUKIR."

There were Cambridge men on each of the three cruisers, *Aboukir*, *Hogue*, and *Cressy*, which were torpedoed and sunk by German submarines. Midshipman C. A. G. Cooke, son of Mr. Arthur Cooke, M.B., Ch.B. Oxon, F.R.C.S. Eng., a member of the surgical staff at Addenbrooke's Hospital, was on the *Aboukir*; we publish his account this week. The thrilling story which he has given of his experiences shows that he undoubtedly owes his miraculous escape to the fact that he could swim well. Midshipman C. A. G. Cooke appears to have been in the water about forty-five minutes when he was picked up by one of the cutters of H.M.S. *Cressy*. Later he was taken aboard a fishing trawler and then transferred to H.M.S. *Legion*, by which he was landed at Harwich.

### A HOSPITAL CHAIRMAN FOR THE FRONT.

The Chairman of the Torbay Hospital, Torquay, Mr. Robert Kitson, has received a commission in the Territorial Reserve and will join immediately.

### THE HOSPITAL BARGE.

Week by week we have given accounts, with many illustrations, of the different forms of travelling hospitals and ambulances which the war has brought into prominence—the hospital train, the hospital ship, the motor-cycle ambulance. We now learn, through the Paris correspondent of the *Times*, that the Union of the Women of France has inaugurated a service of hospital barges for the wounded. The barges will carry surgeons and nurses, and form floating hospitals which can easily be moved about on the magnificent system of waterways for which Northern France is famous.

The first barge, says the writer, was presented on Wednesday last week, and is named *L'Ile de France*. It will accommodate forty wounded men and two surgeons, and should be able to make the journey from the Front to Paris in less than three days. The hospital barge, at all events, has the advantage of easy, if not rapid, motion, on the relatively unblocked water highways, which are not likely to have great military value, and therefore not so likely to be appropriated or greatly interfered with by the troops of either side.

### MENTAL HOSPITAL STAFFS AND THE WAR.

We are glad to see that the London County mental hospitals have provided their fair quota of men from their staffs who are willing to respond to their country's call in its hour of need. Of the officers employed by the London County Council in its mental hospital work no fewer than 338 have joined various branches of the Service. Of these twenty-six were Naval Reservists, 159 Army Reservists, and twenty-six were serving in the Territorial Force when the Force was embodied. Since the outbreak of war three volunteers have joined the Navy (other than the Royal Naval Volunteer Reserve), 120 have joined the Army, three the Territorial Force, and one the Royal Naval Volunteer Reserve. From the Public Health Department of the Council fifty-six officers have responded to the call. Owing to the increased expenditure consequent on the war it will probably be necessary at the beginning of November to increase the present weekly sum charged for the maintenance of each patient in the London County mental hospitals. The present charge is 11s. 1d. a week, and it will probably be necessary to increase it by at least 3½d. a week.

### THE SALARY OF A DEPUTISING MEDICAL OFFICER.

The amount of remuneration that should be given to an assistant medical superintendent during the enforced absence through illness of the medical superintendent has been discussed by the Wandsworth Board of Guardians. Dr. W. L. Maccormac, the superintendent, was unfortunately ill, and his duties were carried out by his assistant, Dr. C. C. Keates. The finance committee recommended that Dr. Keates should receive a gratuity of £50, this being a very little over half the sum that was due to the officer whose duty he had performed. It was the general practice to pay a deputising officer half the salary of his chief, but objection was raised to the proposal on this occasion. The chief point raised was the largeness of the sum, and the condition of the country through the war. Lieutenant Sanders, R.N., said he knew of some doctors in the Navy who had to be lashed to their places aboard ship, and go without food for forty-eight hours; but even such a story as that failed to convince the majority of the board that they should not grant Dr. Keates the extra £50 for managing the large infirmary at Wandsworth during the absence of his chief. So by seventeen votes to four they decided to ask the Local Government Board to sanction the granting to Dr. Keates of the gratuity of £50.

### RADIUM FOR POOR-LAW INFIRMARIES.

The Wandsworth Board of Guardians some months ago suggested to the Local Government Board that a special radium department should be established, under the control of the Managers of the Metropolitan Asylums Board, for the use of patients in Poor-Law infirmaries in London. A much-belated reply has now been received from the Local Government Board stating that they are of opinion that they cannot expeditiously take any action at the present time of the nature suggested, and pointing out that it is open to the Guardians, on the advice of their medical officer, to make arrangements with any general hospital or special institution to carry out treatment of the kind in question. Mr. D. Grundy said the Guardians of Manchester, Salford, East Manchester, and Prestwick had subscribed to a local fund in order to secure treatment at a local institution for their patients. He thought the Metropolitan Boards of Guardians might do something of a similar nature. The Rev. A. G. Pritchard considered that before they pledged themselves to any course they should ascertain from the medical officer what his opinions were with regard to the value of radium in the treatment of medical cases. Mr. W. H. Smith remarked that personally he had no doubt as to the efficacy of the treatment, for his own little girl had been practically cured by the use of radium. The board, however, naturally were not satisfied with a lay opinion on the matter, and referred it to the medical superintendent of the infirmary for his views.

### THE CEREMONY OF CIRCUMCISION ON THE DAY OF ATONEMENT.

The points at which religion and medicine meet are always interesting and sometimes curious, as the following instance, which has been sent to us by a Jewish correspondent, will show: "A very successful circumcision ceremony has been performed by Mr. Louis Pereira Mendoza—a qualified *Mohel* and a member of the Jewish Initiation Society—who was assisted by Nurse L. Miller, late of the Tottenham Nursing Home. This religious function took place on the greatest day in the Jewish calendar, the Day of Atonement, September 30, 1914, or Tishri 10, 5675, in the Great Synagogue, Duke Street, Aldgate, E.C., in the presence of a large congregation. To lend dignity to the proceedings the head of the Jewish Church, the Chief Rabbi, Dr. J. H. Hertz, was present in the capacity of *Sandac*, or holder of the baby boy during the operation, which, occupying less than one minute for all its details, was attended by every care that surgical and hygienic knowledge demand. Though every Jewish male child should, if he is physically sound, be initiated into the Abrahamic covenant when eight days old, it has always been considered a special honour for members of the Jewish community to be allowed the privilege of taking part in this ceremony if it occurs on this great Jewish fast day, and they go to the extent of 'purchasing' the privilege of holding the child during the operation. Thus many a poor Jewish mother, whose son comes into the world seven days before the Day of Atonement, is made happy in more ways than one, and is able with the money she receives from the *Sandac* and others to replenish her household requirements. An apt name was given to this 1914 baby, for being born on the 'Fast of Gedaliah' (Tishri 3), the child was appropriately named 'Gedaliah.'" We are informed that Mr. Mendoza is the youngest *Mohel* or operator in the Jewish community, being only twenty-two years of age.

### LEICESTER'S POOR RECORD FOR HOSPITAL SUNDAY.

In accordance with a custom which has obtained for many years, St. Luke's Day, October 18, will, with certain exceptions, be regarded as Hospital Sunday in Leicester, Leicestershire, and Rutland, and collections taken up on behalf of the Leicester Royal Infirmary. In commending the appeal to the clergy and churchwardens the Bishop of the diocese writes: "The calls upon the liberality of our people which this terrible war necessitates should not interfere with the liberality to be bestowed on such a work as the Leicester Royal Infirmary, and I feel sure that the appeal will meet with a generous reception." We have previously referred to the fact that while towns like Barnsley, Southampton, Dublin, and Newcastle-on-Tyne contribute on Hospital Sunday upwards of £10 per 1,000 of the population, Leicester ought not to be content with a contribution of less than £3 per 1,000 of the population, and we hope the Bishop's appeal will be re-echoed by the clergy and ministers of the county and result in a liberal return for an institution held in the highest respect not only by the residents of the district but by the hospital authorities all over England. Were Hospital Sunday taken up in Leicester as it ought to be, the one weak spot in its hospital work would be removed. There is a specially valuable chance this year to remove it.

### THE NEW BIRMINGHAM SANATORIUM.

An important addition to the Birmingham institutions is the Sir William Cook Memorial Home for Consumptives, which has now been completed at Romsley Hill. With the end of the present week its full complement of patients is expected, and the 110 beds, 65 of which are for men and 45 for women, will be in occupation. With fifteen acres of open ground and ten acres of pine wood the site on the hill is open and healthful, and the view commanded by the slope on which it is built is one of the most extensive in the district. The buildings have been designed by Mr. F. W. Martin; the medical superintendent is Dr. Peter Allan; and the new sanatorium provides 90 of the 409 beds arranged for under the Birmingham tuberculosis scheme. So large a proportion of the workers are subscribers to the Hospital Saturday Fund that Romsley Hill, the Hospital Saturday Home, receives a full share of them. Indeed, the Sir William Cook Memorial Committee and the Birmingham Hospital Saturday Fund arranged, to start with, that if the funds were raised by the committee the Fund would undertake the management and maintenance of the institution. The scheme was a happy one in that the late Sir William Cook had been for thirty years chairman of the City Health Committee and of the committee of the Hospital Saturday Fund. Moreover, the Fund's wide experience of administering convalescent homes for men, women, and children assured that the new institution would be successful. The original plan, which has been much altered, provided for some fifty beds at a cost of £20,000; as half this was only available one block and one wing were first built, and while efforts to make up the financial difference were being made the introduction of the Government's sanatorium scheme led the promoters of the home to bring it into line with the Birmingham plan for dealing with tuberculosis. Thereupon 100 beds were required, and a Government grant of about one-third of the cost became available. All this has stimulated efforts on its behalf, and of the total cost of £30,000 all but £8,500 has been raised, and even this might have been provided had not the occurrence of war caused the final appeal to be withdrawn. Thus the opening ceremony, which it is hoped will coincide with the paying off of the debt, has been postponed to the spring at any rate.

### WANDSWORTH AND THE WAR.

Dr. Keates, first assistant medical officer, St. James' Infirmary, Balham, S.W., has been accepted for service in "Kitchener's Army." He has been appointed medical officer to the 67th Brigade. Dr. Gyllencreutz, first assistant medical officer, St. John's Infirmary, Wandsworth Union, has also joined the new Army as medical officer. The Guardians of the Wandsworth Union are paying half salaries to all those who have responded to their country's call to arms, and will keep their appointments open until they return. The new military hospital on Wandsworth Common is a great centre of attraction at present, especially on Saturdays and Sundays. In a portion of the grounds, those who are almost fit again are often seen playing football.

### POSTPONEMENT OF THE REVISION OF MEDICAL BENEFIT REGULATIONS.

In a recent memorandum, addressed to Insurance Committees and others, the Insurance Commissioners announce that it was their intention to have modified the Regulations governing the administration of medical benefit as from the end of the current year. In view, however, of the fact that so many panel doctors are on active service, while the medical profession as a whole are very fully occupied with matters other than National Insurance, the Commissioners have decided to postpone any radical alteration of the Regulations until more normal conditions prevail. They propose, therefore, to issue Regulations whereby they will be enabled to alter the existing Regulations at the earliest convenient date, instead of having to wait until the end of the next medical year. They also propose to carry into effect, as soon as may be, the decision to standardise the certificates which the doctors are required to furnish to insured persons for the purpose of claiming sickness and disablement benefits. This alteration is certainly non-controversial so far as the doctors are concerned, as it means that they will be saved the trouble of dealing with as many different forms of certificate as there are approved societies of which their patients are members. There can be no doubt that the relief will be welcome.

### A CHANGE IN THE RULES AT TERRITORIAL HOSPITALS.

The experience gained in the course of recruiting 600,000 men for Kitchener's Army proves that a material number of would-be recruits have been rejected by the medical commissioners on account of minor physical defects—hernia, for example. Arising out of this it has been suggested that if such cases would be accepted for service, subject to surgical treatment, and sent on to the Territorial base hospitals, the majority could speedily be enrolled in Kitchener's Army. The admission of a large number of such cases would need special organisation, of course, but the idea is favoured by the knowledge that very many members of the honorary medical, surgical, and nursing staffs of voluntary hospitals have become attached to the Territorial hospitals. From inquiries we have made, we think there is substance in this proposal, and we would venture to suggest that the rules at present in force at Territorial hospitals should be modified, so as to provide for such cases. The military authorities of the War Office, in conjunction with their medical advisers, are in a position to appraise exactly the value of fitting men, suffering from minor physical defects, for enlistment, and we have reason to express confidence that they will weigh these suggestions on their merits without delay.

### A HOSPITAL FOR THE INDIAN TROOPS.

No Englishman could fail to read with enthusiasm Lord Roberts' appeal on behalf of the Indian Soldiers' Fund, the main object of which is "to provide for our Indian soldiers, who may be wounded or fall sick in this war, a hospital in a warm climate in which they will be able to regain health and strength so as to return either to the Front completely cured or to their own country happy and in good spirits." Warm clothing and other comforts are to be provided by the fund, but interest primarily centres upon the hospital and the site which will be chosen for it. With the approval of the Viceroy of India and the Secretary of State, the St. John Ambulance Association has appointed an influential sub-committee, which includes Sir John Hewett as chairman, Colonel Sir T. Wynne, vice-chairman, Lord Roberts, Lord Curzon, Sir H. Perrott, Chief Secretary of the St. John Ambulance Association, Surgeon-General Sir R. Havelock Charles, with Mr. P. D. Agnew and Mr. E. H. Blakesley as honorary secretaries. The proposal is to establish a hospital of 500 beds at Alexandria and a smaller subsidiary hospital at Marseilles. The Secretary, at 1 Carlton House Terrace, is ready to receive subscriptions to the "Indian Soldiers' Fund."

### CLASSIFICATION OF WORKHOUSE INMATES IN SHROPSHIRE.

Some very interesting and revolutionary proposals for the classification of the workhouses in the county have been made recently by a sub-committee of the Shropshire Joint Poor-Law Committee. The sub-committee was appointed to devise a scheme to provide accommodation for the epileptic and feeble-minded chargeable to the various Unions in the county. Their investigations showed that in the fifteen workhouses in the county, with a total accommodation of 2,727 beds, 1,126 were unoccupied, and that the allocation of any one workhouse to the epileptic and feeble-minded class would be attended with many difficulties, unless a general classification of the inmates was undertaken at the same time. The sub-committee suggest that the workhouses at Clun, Cleobury Mortimer, Wem, and Drayton should be used as receiving houses; those at Oswestry, Wellington, Newport, Bridgnorth, and Church Stretton as hospitals; those at Shifnal, Whitchurch, Madeley, and Ludlow as homes for the aged and infirm; that at Ellesmere as a labour colony; and that at Atcham for the epileptic and feeble-minded. In framing this scheme the sub-committee state that they have carefully considered the transport facilities.

### THE WEAK POINT IN THE SCHEME.

It is obvious that if the scheme is adopted it would be possible to close some of the smaller workhouses, though the sub-committee do not suggest this. It is contemplated that each workhouse would still remain under the control of the local Union. This is a weak point in the scheme. It would certainly be better that the Unions should be combined for the joint administrative control of the institutions, and that the separate Unions should deal only with local relief. The scheme is meeting with a good deal of opposition, and may not materialise; but it is interesting as being the first attempt on the part of a combination of county Unions to deal with the problems of institutional relief on the lines we have often advocated.

### PANEL PRACTITIONERS AND FREE CHOICE OF CHEMIST.

The London Insurance Committee has issued to all medical practitioners on the panel for the County of London a notification that in future it is desirable to abstain from using the formula "Rep. mist." when re-ordering a patient's medicine. As the insured person has the right to go to any registered chemist or corporate body employing qualified dispensers, it is felt that the use of the formula "Rep. mist." may not give him this free choice of chemist, in that these prescriptions can only be interpreted by the holder of the original prescription. There is, too, the further point that the term "Rep. mist." may possibly not refer to the actual medicine the practitioner desires his patient to receive. The chemists are not bound by their agreement with the Insurance Committee to copy out the particulars of the original prescription in full on the order "Rep. mist.," and, in fact, a request for a copying fee in these cases has been refused. The Committee state: "After very careful consideration of all the circumstances the Insurance Committee are of opinion that, in cases where a practitioner on the panel desires to order for a patient on his list the same kind of medicine as that ordered on a previous occasion, the order should be written in full, and that no use should be made of the formula "Rep. mist."" At a meeting of the London Panel Committee on Tuesday last, a letter of protest was ordered to be sent to the Insurance Committee with reference to the above, the terms of which are given in our full report of the meeting on page 70 of this issue.

### INSTITUTIONAL PHARMACISTS' WINTER PROGRAMME.

The Council of the Public Pharmacists' and Dispensers' Association has decided to continue as usual the monthly meetings during the coming winter session. An attractive series of lectures and subjects for discussion of special interest to pharmacists engaged in hospital and institution work have been arranged. The inaugural meeting takes place on Wednesday, October 28, at 8 p.m., at the Association's headquarters, St. Bride Institute, Bride Lane, Ludgate Circus, E.C., when Mr. T. Ashcroft Ellwood, M.R.C.S., L.R.C.P., D.P.H., will lecture on "The Influence of the War on Medicine and Pharmacy." Dr. Ellwood is, in addition to his other qualifications, a qualified pharmacist and an expert chemist, being a Fellow of the Institute of Chemistry. Registered pharmacists (ladies or gentlemen), whether members of the Association or not, are welcomed to this opening meeting. The Hon. Secretary is Mr. Geo. W. Gibson, M.P.S., St. Pancras South Infirmary, N.W.

### ALEXANDRA DAY IN LEICESTER.

The final accounts in connection with the organisation of Leicester Alexandra Day have now been presented, and on Wednesday last week the Distribution Committee, consisting of three representatives from each of the charities benefiting in the division of this year's effort, met to decide the claims of each institution. It was explained that the receipts amounted to £2,618, including £614 (less expenses £82) received from the Mayor's Alexandra Day Fund organised by Councillor J. R. Frears, and after expenses of flowers, advertising, etc., were deducted there was a balance of £2,000 for division. The President of the Leicester Cripples' Guild, Mr. Arthur I. Groves, who was responsible for the organisation of the effort, presided, and entire satisfaction was shown with the following awards: The Leicester Cripples' Guild, £500; the Leicester Children's Hospital, £450; the Leicester Poor Boys' and Girls' Summer Camp, £380; the Leicester Institution for the Blind, £300; the Leicester District Nurses' Association, £300; the Surgical Aid Society (Leicester Branch), £70. It was resolved to extend the date for receiving applications from societies desirous of sharing in next year's proceeds to December 31 next.

### CHOCOLATE AND TOBACCO AS CHURCH DECORATIONS.

Previous to the date arranged for the Harvest Festival services at St. Luke's Church, Lyncombe, Bath, the vicar, the Rev. C. E. Doudney, announced to the congregation that this year, in consequence of the war, the usual church decorations would be dispensed with and be superseded by gifts for the soldiers, with the result that the church on the day of the Harvest Thanksgiving presented a remarkable appearance, the east end being filled with piles of blankets, rugs, tinned delicacies, chocolate, tobacco and cigarettes, which had been sent in by the parishioners and members of the congregation. These are to be distributed to the hospitals and soldiers.

### A SEPTEMBER RECORD.

With two military hospitals at Whitworth Street and Princess Street, and a number of beds which have been placed at the disposal of the War Office by the Manchester Royal Infirmary and Salford Royal Hospital, the committee of management of Ancoats Hospital, Manchester, have felt that, considering the limited number of beds at their disposal, only 114, and the great demand constantly being made upon them, their first duty is to attend to the civil population. Their resolve appears justified from the record of work done during September, when 152 patients were admitted to the wards, the average daily number of beds occupied being 107.3; the number of new out-patients was 769, with 4,136 attendances; while the number of new accident cases treated totalled 1,696, of whom twenty-one were so seriously injured as to call for immediate admission.

### A MANCHESTER HOSPITAL AND THE WAR.

Ancoats Hospital has had the privilege of sending for war service two surgeons from the honorary staff, Mr. W. R. Douglas and Mr. John Morley, and one physician, Dr. J. F. Ward, who have left for unknown destinations with the Territorial Forces. Six sisters have gone to the staff of the military hospitals, five having been called to the Manchester hospitals—namely, Miss Allen, Miss M. Jones, Miss E. E. Jones, Miss Pickett, and Miss E. A. White—while Miss Bell has joined her Territorial branch at Cambridge. In the accident rooms of the hospital two Red Cross nurses attend for three hours each morning and two for two hours each afternoon for practical training, while Miss L. H. Beard, the matron, who takes a keen interest in the East Lancashire branch of the Red Cross Society, has voluntarily undertaken to teach a limited number of their nurses in practical bedside nursing, and has already held three or four different classes for two hours three times a week.

